# Multiple cholangiocarcinomas in the intrahepatic and extrahepatic biliary tree due to dichloromethane exposure: a case report

**DOI:** 10.1186/s40792-020-00842-9

**Published:** 2020-04-21

**Authors:** Daisuke Ogawa, Hiromitsu Hayashi, Fumimasa Kitamura, Norio Uemura, Tatsunori Miyata, Hirohisa Okabe, Katsunori Imai, Yoichi Yamasita, Shoji Kubo, Hideo Baba

**Affiliations:** 1 Department of Gastroenterological Surgery, Graduate School of Life Sciences Kumamoto University, 1-1-1 Honjo, Kumamoto, 860-8556 Japan; 2 Department of Hepato-Biliary-Pancreatic Surgery, Osaka City University, Osaka, Japan

**Keywords:** Multiple cholangiocarcinoma, Multicentric cholangiocarcinoma, Occupational cholangiocarcinoma, Dichloromethane, 1,2-Dichloropropane

## Abstract

**Background:**

An outbreak of cholangiocarcinoma in Japan has led to widespread concern among workers in printing plants. In March 2013, the Japanese Ministry of Health, Labour and Welfare, confirmed a causal relationship between cholangiocarcinoma and long-term exposure to dichloromethane (DCM) and 1,2-dichloropropane (DCP), which were widely used in printing plants. We herein report a rare case of successful radical resection of multiple cholangiocarcinomas in the intrahepatic and extrahepatic bile ducts caused by past exposure to DCM.

**Case presentation:**

A 54-year-old man developed brown urine 22 years after his last exposure to DCP and DCM. He had an 11-year history of working at a printing plant from the age of 21 to 31 years and dealt with organic solvents during his employment. Enhanced computed tomography revealed a thickened distal bile duct wall with upstream biliary dilatation and multiple intrahepatic cholangiocarcinomas located in liver segments III, VI, and VIII. Biopsy of the distal bile duct wall revealed adenocarcinoma, and a diagnosis of distal cholangiocarcinoma was made. Tumor marker levels were within the reference range (carcinoembryonic antigen, 3.3 ng/mL; carbohydrate antigen 19-9, 25.4 U/mL; SPAN-1, 13 U/mL; and DUPAN-2, 33 U/mL). The multiple intrahepatic and extrahepatic bile duct cancers were treated by subtotal stomach-preserving pancreatoduodenectomy and partial hepatectomy of segments III, VI, and VIII. Pathological examination of the surgical specimens revealed multiple cholangiocarcinomas with well-differentiated adenocarcinoma in the biliary tree. The patient was still alive without recurrence 17 months after the operation.

**Conclusions:**

We experienced a rare case of multiple cholangiocarcinomas in the intrahepatic and extrahepatic bile ducts that developed 22 years after the patient’s last exposure to DCP and DCM. Long-term and careful follow-up is required for workers with an occupational history of exposure to organic solvents because of the risk of development of cholangiocarcinoma.

## Background

A cholangiocarcinoma outbreak occurred at a printing company in Osaka, Japan [1–3]. Dichloromethane (DCM) and 1,2-dichloropropane (DCP) played a key role in the development of this type of cholangiocarcinoma [4]. Long-term exposure to high concentrations of DCM and/or DCP is epidemiologically associated with the development of biliary tree cancers [5], the underlying molecular mechanism is still unclear. In the whole-exome analysis of occupational cholangiocarcinoma cases, a higher mutation burden in the invasive carcinomas (mean 76.3/Mb) and precancerous lesions (mean 71.8/Mb) of the occupational cholangiocarcinoma cases than in the lesions (mean 1.6/Mb) in the non-occupational cholangiocarcinoma cases [6]. In 2013, the Japanese Ministry of Health, Labour and Welfare, initially recognized this type of cholangiocarcinoma as an occupational disease. Several cases of occupational cholangiocarcinoma have been reported to date. We herein describe a patient who was diagnosed with multiple cholangiocarcinoma 22 years after his last exposure to high concentrations of DCP and DCM while working at a printing company in Nagoya city.

## Case presentation

**Fig. 1:**
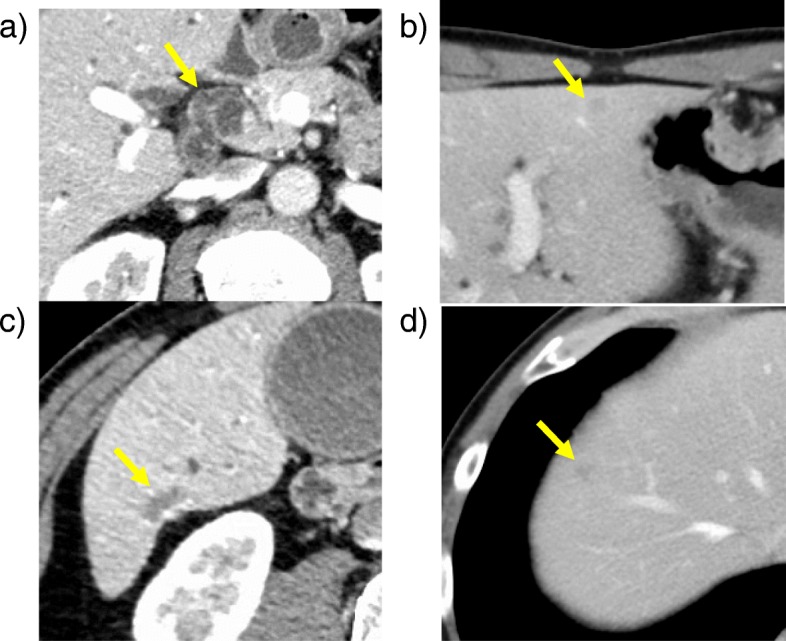
Multiple intrahepatic and extrahepatic bile duct cancers shown by dynamic abdominal computed tomography. **a** Thickening of the distal bile duct wall with upstream biliary dilatation (arrow). Multiple low-density tumors with peripheral enhancement in **b** segment III, **c** segment VI, and **d** segment VIII in the liver (arrow)

**Fig. 2:**
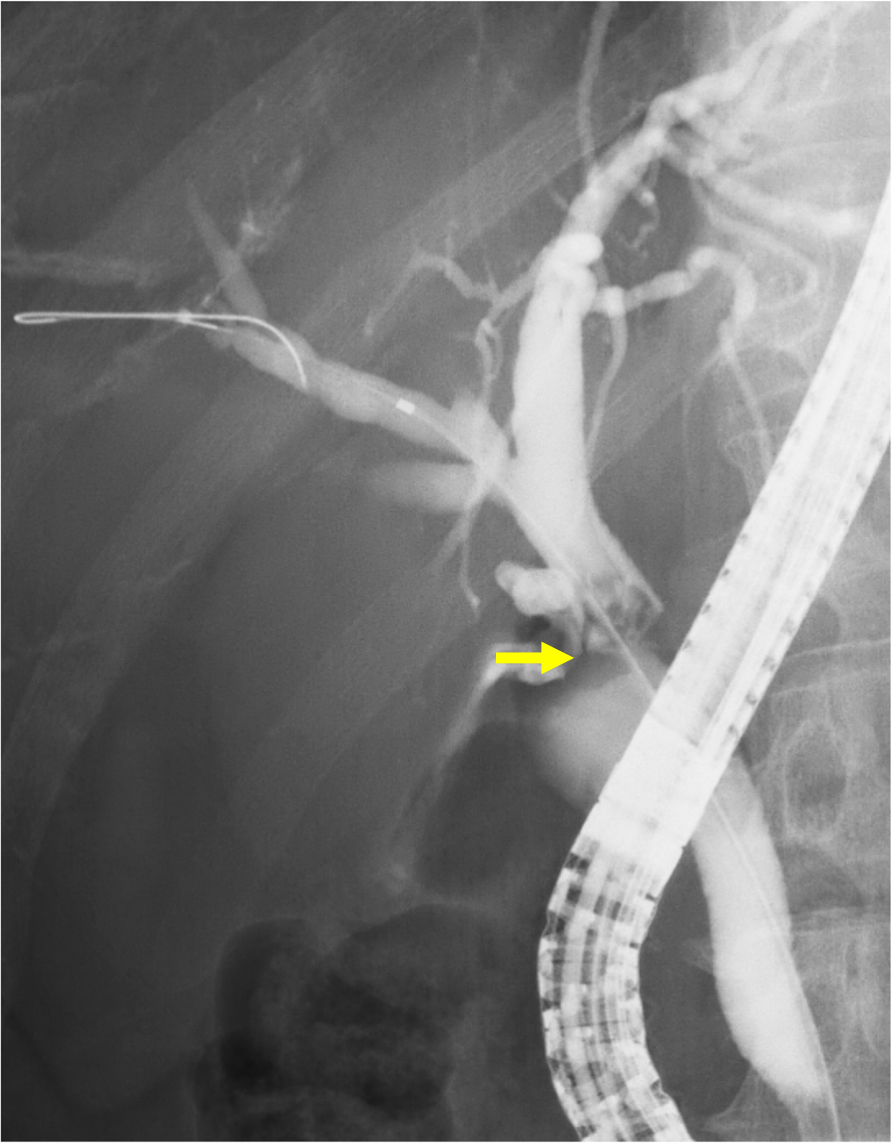
Endoscopic retrograde cholangiopancreatography revealed stenosis of the distal bile duct. Examination of a biopsy specimen from the distal bile duct wall revealed adenocarcinoma (arrow)

A 54-year-old man presented for evaluation of brown urine. He had an 11-year history of working at a printing company from the age of 21 to 31 years, with exposure to high concentrations of DCM and DCP during his employment. He had no medical history. Laboratory tests revealed elevated levels of serum total bilirubin (7.0 mg/dL), aspartate aminotransferase (227 U/L), alanine aminotransferase (417 U/L), gamma-glutamyl transferase (1174 U/L), and alkaline phosphatase (1653 U/L). The serum concentrations of carbohydrate antigen 19-9 and carcinoembryonic antigen were within the normal range (25.4 U/mL and 3.3 ng/mL, respectively). Dynamic computed tomography showed a circumferential tumor of the distal bile duct with upstream biliary dilatation (Fig. 1a) and multiple low-density tumors with peripheral enhancement at liver segments III, VI, and VIII (Fig. 1b–d). Gadoxetic acid-enhanced magnetic resonance imaging showed similar findings. Adenocarcinoma cells were detected in the distal bile duct tumor by examination of a biopsy specimen obtained using endoscopic retrograde cholangiopancreatography (Fig. 2). From these laboratory data, radiological findings, and history of working at a printing plant, the patient had the possibility of multiple and multicentric intrahepatic and extrahepatic cholangiocarcinomas. We explained the ideal surgical approach to the patient and his family, and they consented to surgical removal of the multiple bile duct cancers.

**Fig. 3:**
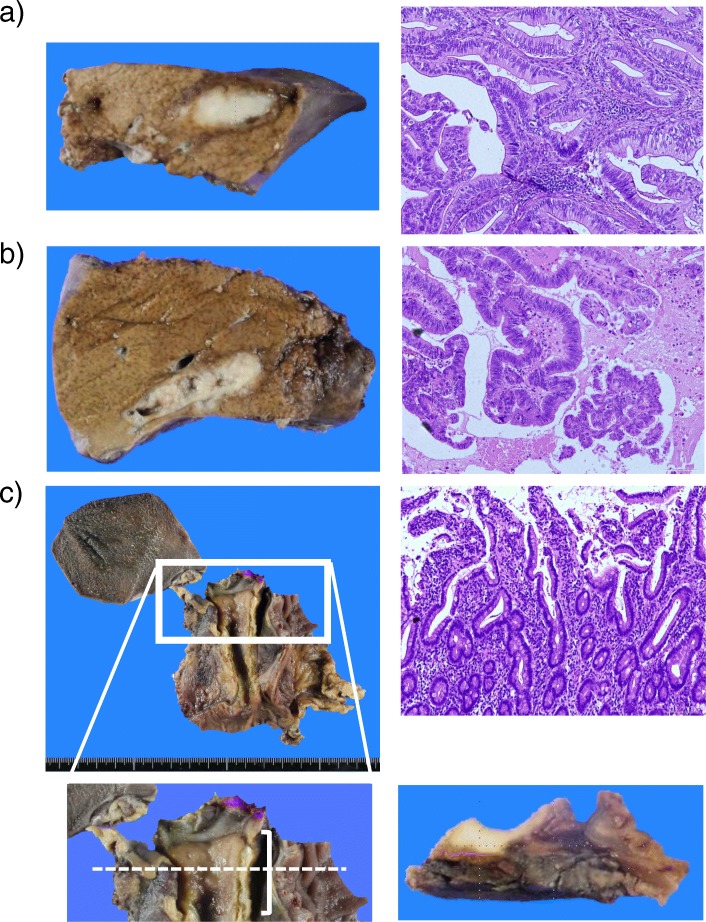
Pathological findings of the intrahepatic and extrahepatic bile duct tumors. **a** Intrahepatic cholangiocarcinoma with well-differentiated adenocarcinoma, 1.9 cm (segment III), macroscopic image (left panel) and microscopic image (right panel). **b** Intrahepatic cholangiocarcinoma with well-differentiated adenocarcinoma, 2.8 cm (segment VI), macroscopic image (left panel) and microscopic image (right panel). **c** Distal cholangiocarcinoma with well-differentiated adenocarcinoma, macroscopic image (upper left panel), microscopic image (upper right panel), image magnification (lower left panel). The bracket indicates the range of extrahepatic bile duct tumor, cross-sectional image at the dot line (lower right panel)

One month after the initial visit, subtotal stomach-preserving pancreatoduodenectomy, subsegmentectomy (segment VI), and partial hepatectomy (segments III and VIII) were performed. The operation was completed with no major problems. Pathological examination revealed intrahepatic cholangiocarcinoma with well-differentiated adenocarcinoma, periductal infiltrating type (both of segments III and VI), 2.8 cm (segment VI), 1.9 cm (segment III), ig, fc(−), fc-inf(−), sf(−), s0, ne0, vp1 (segment III), vv0, va0, b1 (both of segments III and VI), sm(−), ch (a1/f1), pT4, pN0, and no neoplastic changes in segment VIII (Japanese rule 6th edition) (Fig. 3a, b) and distal cholangiocarcinoma with well-differentiated adenocarcinoma (Bp, nodular-infiltrating type, circ, 21 mm, tub1 > tub2, pT2a (SS), int, INFb, ly1, v1(VB), ne2, pN0(0/38), pPM0, pDM0, pEM0, pPV0, pA0, R0) (Japanese rule 6th edition) (Fig. 3c). AJCC staging of intrahepatic cholangiocarcinoma (AJCC/UICC 8th edition) was grade 1, pT2, and pN0, and AJCC staging of extrahepatic cholangiocarcinoma (AJCC/UICC 8th edition) was grade 1, pT2a, and pN0, respectively. As for the resected specimen of segment VIII, there was no pathological finding related to the false-positive radiological image like a cholangiocarcinoma. Additionally, in the background liver, activity and fibrosis were A1 and F1, respectively. There were no findings of chronic bile duct injury, proliferative changes, and lesion of biliary intraepithelial neoplasm (BilIN) by pathological assessment. To assess the genetic characteristics in each tumor, the intratumoral methylation levels of long interspersed nucleotide factor-1(LINE-1), which is regarded as a surrogate marker of global DNA methylation [7], were measured by laser micro-dissection and bisulfite genomic sequencing in each tumor (intra- and extra-hepatic cholangiocarcinomas) as previously described [8, 9]. The methylation levels of intrahepatic and extrahepatic cholangiocarcinomas (segment III, segment VI, and extrahepatic hepatic duct) were 75.23 ± 4.42, 85.88 ± 1.9, and 82.34 ± 3.11, respectively. After the operation, the patient underwent 6 months of adjuvant chemotherapy with oral S-1 (a fluoropyrimidine). He was still alive 17 months after the operation with no recurrence.

## Conclusions

According to previous studies, the characteristics of occupational cholangiocarcinoma include elevated serum gamma-glutamyl transferase activity, regional dilatation of the bile duct without tumor-induced obstruction and biliary findings similar to those of primary sclerosing cholangitis on diagnostic imaging, the presence of precancerous or early cancerous lesions, and nonspecific bile duct injuries [1, 3, 5, 10–12]. Other suggested characteristics are multicentric and multistep carcinogenesis with a high frequency of somatic mutations secondary to chronic bile duct injury and precancerous or early cancerous lesions [6, 11, 13]. Moreover, a previous study reported that only two cases of multiple intrahepatic and extrahepatic occupational cholangiocarcinoma with radical resection have been described. The pathological findings in the present case revealed three malignant adenocarcinoma lesions developing in the intrahepatic and extrahepatic bile ducts. Dilatation of the intrahepatic bile ducts with tumor-induced obstruction was apparent in this case. No other characteristics, such as precancerous lesions, were present. Occupational cholangiocarcinoma is certified by the Japanese Ministry of Health, Labour and Welfare, and our patient was also identified as occupational cholangiocarcinoma due to his 11-year history of exposure to high concentrations of DCM and DCP at the printing company where he worked. In a previous study, the longest period between the end of exposure to such organic solvents and the diagnosis of occupational cholangiocarcinoma was 18 years [14]. In the present case, cholangiocarcinoma was diagnosed 22 years after the last exposure; therefore, this is the longest period between the last exposure and cholangiocarcinoma development among Japanese patients diagnosed with occupational cholangiocarcinoma to date. Careful long-term follow-up is thus necessary for workers who are exposed to such organic solvents because of the potential for cholangiocarcinoma development even after the end of exposure. Furthermore, in the present case, the radical resection for multiple cholangiocarcinomas in the intrahepatic and extrahepatic biliary tree could provide a favorable prognostic outcome as in the previous reports [1, 11]. In this case, it is difficult to exclude the possibility of intrahepatic metastasis from extrahepatic biliary duct cancer. In light of the different methylation levels in the intrahepatic and extrahepatic cholangiocarcinomas, the clinicopathological findings and favorable prognostic outcome provide the possibility of the multicentric developments in the intrahepatic and extrahepatic biliary tree due to the past exposure to DCP and DCM as in the previous reports [5, 11]. The radical resection for the multicentric cholangiocarcinomas in the intrahepatic and extrahepatic biliary tree due may lead to favorable prognostic outcomes [5]. As for adjuvant chemotherapy for resected biliary tract cancer, there is still no solid evidence. BCAT study (adjuvant gemcitabine versus surgery alone) and PRODIGE 12 study (adjuvant gemcitabine and oxaliplatin versus surgery alone) could not show prognostic improvement in extrahepatic bile duct cancer [15, 16]. BILCAP study (adjuvant capecitabine versus surgery alone) showed the superiority of adjuvant chemotherapy only in a per-protocol set, but not in an intention to treat analysis [17]. Additionally, capecitabin is not available (not covered by health insurance) for biliary tract cancer in Japan. Adjuvant chemotherapy with oral administration of S-1 after hepatectomy for biliary tract cancer improved prognostic outcomes compare to gemcitabine at phase II study (KHBO1208). Now, ASCOT study (adjuvant S-1 versus surgery alone, phase III study) has been ongoing for resected biliary tract cancer since 2013 [18]. We explained to him and his family that there was no clear evidence of adjuvant chemotherapy in multiple and resected biliary tract cancer, and it is limited in case series [19]. Then, he and his family desired to take an oral S-1 as an adjuvant chemotherapy. We would wait for the results of the ASCOT study and ACTICCA-1 study (gemcitabine and cisplatin versus surgery alone) [20] to elucidate the clinical benefits of adjuvant chemotherapy for biliary tract cancer.

In summary, we experienced a rare case of multiple cholangiocarcinomas in the intrahepatic and extrahepatic biliary tree that developed 22 years after the last exposure to DCP and DCM. Long-term and careful follow-up is required for workers with an occupational history of exposure to high concentrations of DCM and/or DCP because of the possibility of the development of cholangiocarcinoma. Finally, to ensure an accurate diagnosis of occupational cholangiocarcinoma, not only laboratory data and radiological findings but also a confirmed history of working at a printing plant is very important.

## Data Availability

All datasets presented in the main paper are available whenever possible.

## Abbreviations

DCM: Dichloromethane
DCP: 1,2-Dichloropropane
